# Editorial

**Published:** 1872-11

**Authors:** 


### EDITORIAL.

### State Board of Dental Examiners.

The annual session of the Board will be held in Columbus, at the Neil House, on Tuesday, the 3d of December next, beginning at 10 o'clock A. M.

It is very desirable that all who intend to meet with the Board should be as prompt at the time specified as possible, that the work may proceed without delay. It is desirable to finish the examination as nearly as possible on that day and evening, as the State Dental Society meets on the following day, and the way should be clear for that.

### Ohio State Dental Society.

The seventh annual meeting of this society will be held in the city of Columbus, beginning on Wednesday, the 4th of December next, at 10 o'clock, A. M., in Odd Fellows' Hall. At which time and place we hope every member of the society will be present, and fully prepared for the work to be done. Let every one resolve that the meeting shall be better for his being there. We trust that not only will every member be there, but that every dentist in the State, who has at heart the good of his profession, will make it a point to be present.

There is much important work to be done at this meeting—work that interests the profession and the public. Remember that the influence and results of the earnest, persistent, intelligent worker, are incalculable. Then let *all* come, and each one *prepared* for *work.*

### The Dental Law.

The probationary period of the law regulating the practice of dentistry in the State of Ohio, terminates on the last day of December next—two months from this time. It is presumable that all interested have made note of this fact, and will govern themselves accordingly. Many in the State have already done so, and doubtless a large proportion of the remainder will do so before the first of January.

It would be highly gratifying to know that *all* practicing dentists within the State had conformed to the requirements of this law, which is admitted by all to be a good one. None will claim that incompetent persons should practice so important and responsible a calling as dentistry—a work intimately connected with and influenciug the comfort, health, and well-being of a large proportion of the people in the community.

Every one having any just appreciation of the value and importance of the teeth, and knowing that there is a large number in the dental profession, in active practice, who are totally incompetent for the proper performance of its duties, will fully appreciate the justice and importance of this law. The people as well as the dentists are interested in the faithful carring out of the provisions and requirements of the law.

### Tennesse State Dental Society.

It was our privilege to attend the recent meeting of this society. It was not largely attended, but those present were very enthusiastic in their interest and efforts for the advancement of the profession.

Several excellent papers were read, and the discussions upon them and the various subjects presented, were very interesting and instructive.

At a former meeting a committee was appointed to prepare a report that should embrace a draft of a popular essay, which if approved, should be published and furnished to the members of the society for distribution in their respective fields of operation. The committee failed to report; but a volunteer report had been prepared, which was presented and read. The paper was so good that with a few changes that were suggested, it was unanimously approved, and the Publication Committee was directed to publish it in a proper form for the use of the profession. The essay was one of the best we have ever seen in this direction. Its distribution by every dentist would be the occasion of much good to both the profession and the public.

We should be glad to see every society in the country engage in work as deffinite and effectual as this. We see no reason why this should not be done, the field is great and the demand very urgent.

There are many good members of the profession in Tennessee whom we expected to meet there, and whose absence caused disappointment to all who were present; doubtless each one imagined he had a good excuse for his absence; but it is rather singular that so many should be hindered at one time, if there was the earnest desire to do good and receive good, that should be in every truly professional man's heart; still we cannot fault any one.

### Causes of Necrosis.

DR. TAFT, *Dear Sir:* Some five months ago, a man came to me with the lower jaw, left side, badly swoolen, and on examination I found the first molar tooth broken off, and the roots just visible above the gums, with an opening outside and between them, and the second bicuspid discharging pus copiously. The second bicuspid was dead and very loose. He had on the previous Thursday (this was Sunday) gone to a physician some fifty miles away to have it extracted, who only lanced it and sent him to me. I removed the bicuspid which was followed by a discharge of pus through the opening. The roots were very difficult to remove, being badly exostosed. I saw nothing more of him for two months, although I had requested him to call again if he did not get relief. The disease was not arrested, and he is now commencing a suit against me for malpractice. Now I wish to ask, did you ever know the process fractured by extracting the *lower bicuspids*, or detached roots of the molars? Is not necrosis (of the jaw) a frequent *result of ulceration about the teeth or roots?* Do you not often extract sound teeth that are dead and ulcerated? How *soon will exfoliation begin* after necrosis of the bone takes place? Is not exfoliation the usual result of necrosis? These questions, Doctor, I would be glad if you would answer plainly, as he alleges that I fractured the alveolar process while extracting the bicuspid, and that that produced necrosis of the bone—notwithstanding they admit that they went the Thursday before to have them removed by another party. In my practice of over twenty years I do not recollect now to have seen any process removed with the lower bicuspids. Yours truly,

M. L. J.

I have never known the alveolus fractured by the removal of an inferior bicuspid, nor by the extraction of the roots of inferior molars after their separation; there is little or no probability that in either case such a result would occur.

Necrosis very frequently accompanies or follows ulceration, or suppuration, from the roots of the teeth.

Teeth that are not decayed have sometimes to be removed, in consequence of the disease of the surrounding parts, or from the destruction of the parts that support them.

After necrosis, exfoliation varies much in time and manner of its occurrence, sometimes, in good constitutions, it is definitely effected in a short time, in others--unfavorable cases--there will be no exfoliation; but usually the dead part is thrown off from the living.

The mere fracture of the alveolus, or even breaking off a portion of it, will not produce necrosis of that which remains.

### Bibliographic.

THE PATHOLOGY OF THE TEETH; with special reference to their Anatomy and Physiology. By Carl Wedl, M. D., Professor of Histology in the University of Vienna. Translated from the German by W. E. Boardman, M. D.; with notes by T. B. Hitchcock, M. D., D. M. D.; Professor of Dental Pathology and Therapeutics in Harvard University. With one hundred and five illustrations. Published by Lindsay and Blakiston, 1872.

The want of such a work as this has long been felt by the dental profession. There has hitherto been no systematic work upon the pathology of che teeth, coming up to the present attainments upon the subject.

The work consists of two general divisions: first, Anatomy and Physiology; and second, Pathology; apresentation of the former being necessary for the proper elucidation of the latter. There has been no work presented to the profession that so thoronghly occupies the ground as this, which gives special attention to most if not all the diseases and abnormal conditions that attach to the teeth; discussing each fully and clearly; dealing with facts rather than hypotheses.

This work should be in the hands of every dentist, and receive a thorough examination. The work deals with many plain, palpable things, of great importance, that have hitherto been almost wholly ignored.

We advise every one to procure the work, and then study it wholly and thoroughly.

### Obscure Case.

BY H. SCOTT.

A young man of twenty years came with his mother to have his teeth filled. The first one he wished filled was one that was aching. He pointed out the second left inferior bicuspid. It had ached "*coming along*," he said. It was entirely sound in every respect, except the smallest decay on the grinding surface.

His upper incisors were more or less decayed, the left lateral considerably. I examined them with probe; and when I had passed it down into the left lateral, firmly, the lower bicuspid began to ache so severely as to bring tears from his eyes, and cause him to writhe in his seat. Creosote applied to the incisors brought instant relief to the bicuspid. I subsequently devitalized the upper nerve, and that was the last of the case.

Such cases are of course common to the profession, and I only communciate this to admonish those of less experience to be careful of diagnosis before acting in obscure cases. A fatal error would have been the extraction of the tooth the man complained of; a thing that is often done by the inexperienced.

